# The impact of artificial intelligence on sustainable development performance of public healthcare supply chain-an empirical study based on listed Chinese pharmaceutical companies

**DOI:** 10.3389/fpubh.2025.1549283

**Published:** 2025-07-23

**Authors:** Qianlan Chen, Ping Long, Ruihai Dong, Chaoling Li, Siyi Mao

**Affiliations:** ^1^School of Economics and Management, Guangxi Normal University, Guilin, China; ^2^China Petroleum and Chemical Corporation, Shandong Petroleum Branch, Jinan, China; ^3^Key Laboratory of Digital Empowerment Economic Development, Guangxi Normal University, Guilin, China

**Keywords:** artificial intelligence, public healthcare supply chain, sustainable development performance, green supply chain management practice, industry competition

## Abstract

With the continuous deepening of the digital transformation of the public healthcare supply chain, as the next step of digitalization, intelligent transformation has received much attention, especially how artificial intelligence can improve the sustainable development performance of the supply chain needs to be studied. In order to fill this gap, this paper establishes a research model of artificial intelligence, green supply chain management practice, industry competition and sustainable development performance of public healthcare supply chain. Taking Chinese listed pharmaceutical enterprises as research objects, this paper conducts empirical analysis on 301 valid questionnaires through questionnaire survey and structural equation model. The relationship between artificial intelligence, green supply chain management practice, industry competition and sustainable development performance of public healthcare supply chain is studied. In this paper, green supply chain management practice is regarded as the intermediary variable, industry competition is regarded as the moderating variable, and a research model with both mediation and regulation is constructed. The empirical results show that: (1) Artificial intelligence has a significant positive impact on the sustainable development performance of public healthcare supply chain; (2) Artificial intelligence has a positive impact on green supply chain management practice; (3) Green supply chain management practice has a positive impact on the sustainable development performance of public healthcare supply chain; (4) Green supply chain management practices play an intermediary role in the effect of artificial intelligence on the sustainable development performance of public healthcare supply chain, that is, artificial intelligence improves the sustainable development performance of supply chain through green supply chain management practices; (5) Industry competition actively regulates the impact of artificial intelligence on the sustainable development performance of public healthcare supply chain. This study enriches the literature on AI and public healthcare supply chains, while providing new practice guidelines for sustainable development and modern management of public healthcare supply chains.

## 1 Introduction

In the post-pandemic era, economic instability, climate change, and the energy crisis are putting enormous pressure on public health systems (1) and exacerbating inequalities in health and access to health services. However, this has also accelerated the innovation of modern public healthcare supply chain in terms of development, distribution and technology application (2), and the study of public healthcare supply chain has become a hot spot in academia and industry. The public healthcare supply chain plays a key role in the realization of social healthcare, but the global health and medical industry still faces health, economic and environmental challenges, which highlight the important position of the public healthcare supply chain in responding to public health events, and also generate the need to study the sustainable development performance of the public healthcare supply chain and use modern technological tools (3).

At the same time, the application of artificial intelligence in the field of public healthcare has become an increasingly popular trend (4). First of all, artificial intelligence can optimize the management of public healthcare supply chain, drive the management model to be more intelligent, to adapt to the complex and changing market environment, and improve the resilience of the supply chain. Second, AI can help public health systems warn of epidemic threats through big data analysis, thereby helping to prevent the spread of infectious diseases and mitigate public health crises. Third, artificial intelligence has obvious advantages in providing decision support. Artificial intelligence can deal with the problem of information overload (5), help decision makers filter all possible options, and recommend the most appropriate scheme for a specific situation.

All in all, with the in-depth application of artificial intelligence in the field of public health, it is expected to strengthen disease prevention and population health management, achieve sustainable development of the public healthcare supply chain, and make outstanding contributions to promoting the development of global public health. The existing literature provides useful insights and methodological support for understanding the impact of artificial intelligence on the public healthcare supply chain. However, there are still two shortcomings in the existing research: (1) There are few literatures directly studying the impact of artificial intelligence on the sustainable development performance of the public healthcare supply chain. Most existing scholars have discussed the impact of digital capability on sustainable performance. Some scholars believe that the improvement of digital capability can optimize resource allocation, improve operational efficiency, and improve sustainable performance (6, 7). Another part of scholars believe that digitalization requires a large amount of investment and risks of data leakage, which is not conducive to the improvement of sustainable performance (8, 9). (2) Research on the impact of artificial intelligence on the sustainable development performance of public healthcare supply chain needs to be deepened and expanded. The existing literature lacks in-depth analysis of the effect path of AI on the sustainability performance of public healthcare supply chain. Most of the papers focus on verifying the role of a certain variable between AI and healthcare supply chain (10), while ignoring the process of AI affecting the sustainability performance of public healthcare supply chain through these variables and the causal relationship between these variables. Therefore, in order to fill this gap, based on resource-based theory, dynamic capability theory and sustainable development theory, this paper focuses on the impact of artificial intelligence on the sustainable development performance of public healthcare supply chain. In addition, the paper further discusses the mediating role of green supply chain management practice and the moderating role of industry competition.

Since the mechanism of action is not clear in previous studies, this study has three main contributions. First, we address an overlooked and understudied question from previous research: Will AI have a positive effect on the sustainability performance of public healthcare supply chains? Previous studies have focused on the impact of digital capabilities on sustainability performance, and there is controversy about whether digital capabilities can improve sustainability performance, but we directly demonstrate that artificial intelligence positively affects sustainability performance. Secondly, we take green supply chain management practice as a mediating variable to further understand the impact and role of artificial intelligence on the sustainable development performance of public healthcare supply chain. Finally, with industry competition as a boundary condition factor that defines AI innovation, we introduce industry competition as a moderating variable to illustrate the role of industry competition in the relationship between AI and sustainability performance. By exploring the impact of AI on the sustainability performance of public healthcare supply chains, we provide valuable insights and practical guidance for the application of AI in the sustainability of public healthcare supply chains.

## 2 Research hypothesis

### 2.1 Artificial intelligence and sustainable development performance of public healthcare supply chain

The public healthcare supply chain is now facing many challenges, including the continuous iteration of technology and the intensification of market competition, which forces the supply chain to carry out intelligent transformation in order to maintain a competitive advantage. The key to improve the core competitiveness of supply chain is to have unique and difficult to replicate resources and capabilities, which conforms to the theory of resource-based view. As a new economic form, digital economy has increasingly become an important engine to promote high-quality economic development (11, 12), and new products, new formats and new business models based on digital technology have become an important force to promote global economic growth (13). As a core research achievement in the field of digital economy, artificial intelligence has gradually developed into an important driving force for a new round of technological revolution and industrial change. Artificial intelligence provides innovative methods for the development of supply chain (14), and its recommendations based on multiple algorithms and data models provide higher recommendation quality than traditional recommendation methods (15), which can analyze and solve some problems that cannot be addressed by medical technology (16). Artificial intelligence technology promotes the development of smart medical treatment. The feasibility of its application in the public healthcare field has been confirmed, and the future prospect is broad (17). Through data analysis, artificial intelligence can intelligently generate important strategies and suggestions to help managers make relevant predictions (18), ensure the timely provision of pharmaceutical products and services, enhance the ability of the public healthcare supply chain to respond to public health emergencies and rapidly deploy key resources, and prevent potential risks. In addition, AI helps optimize the logistics network of the public healthcare supply chain, which can help the supply chain save costs and minimize waste.

According to relevant studies, artificial intelligence can make full use of data and information through the application of digital technology and resource reset, reduce the complexity and uncertainty of information, and ultimately achieve sustainable development of organizations (19). Specifically, in terms of economic sustainability, the inclusion of artificial intelligence technology in the public healthcare supply chain can better integrate information resources, break the information barriers between different subjects, and realize real-time information sharing (20, 21). In this way, it can sense changes in user needs in time, provide customers with solutions and products in time, and thus gain market competitive advantages. Achieve sustainable growth of economic benefits (22). In terms of social sustainability, the application of artificial intelligence technology in the public healthcare supply chain means that the production efficiency of the supply chain is high (23) and the operation of employees is more standardized, which is conducive to preventing unsafe situations, providing a safer operating environment for employees and safer medical products for the public. Secondly, the inclusion of artificial intelligence in the management process of public healthcare supply chain helps to avoid individual decision-making mistakes and protect the rights and interests of relevant stakeholders. In terms of environmental sustainability, AI can minimize waste (24), improve cost-effectiveness, and ensure the timely supply of critical materials (25). Secondly, artificial intelligence algorithms can optimize the logistics of the healthcare supply chain, improve transportation efficiency, reduce fuel consumption, and minimize carbon emissions, thus promoting the sustainable development of the public healthcare supply chain environment. Therefore, this paper believes that artificial intelligence has a positive effect on the sustainable development performance of public healthcare supply chain. Based on this, this paper puts forward the following hypothesis.

Hypothesis 1: Artificial intelligence positively affects the sustainable development performance of public healthcare supply chain.

### 2.2 Artificial intelligence and green supply chain management practice

With the global attention to environmental protection and sustainable development, green supply chain management has become a key path for enterprises to achieve sustainable development. At the same time, the booming development of artificial intelligence has brought new opportunities and changes to green supply chain management practices. The practice of green supply chain management requires enterprises to fully consider environmental protection and resource utilization efficiency in all aspects of the entire supply chain process, from raw material procurement, manufacturing, product distribution, product use and recycling (26), so as to reduce the negative impact on the environment and achieve the coordinated development of economy, society and environment (27). As a cutting-edge technology with powerful data processing and analysis capabilities and intelligent decision-making functions, artificial intelligence is gradually penetrating into all aspects of green supply chain management practices. Specifically, first of all, regarding the evaluation and selection of enterprise suppliers, artificial intelligence technology can be used to analyze and evaluate the product quality, environmental performance and social responsibility of suppliers (28), establish a supplier evaluation model, and help enterprises choose suppliers that are more in line with the requirements of green supply chain management practices. Secondly, artificial intelligence can be used in the green production of the supply chain (29). Enterprises can use artificial intelligence technology to monitor and analyze data in the production process in real time, optimize production parameters and process flow, improve production efficiency, and reduce energy consumption and waste generation. Finally, artificial intelligence can be used to optimize supply chain transportation routes (30), and combine real-time traffic information, weather conditions, vehicle load and other factors to plan the optimal route for logistics distribution vehicles, reduce transportation mileage and time, and reduce energy consumption and carbon emissions. In fact, in addition to the application of artificial intelligence in the green supply chain management practice mentioned above, artificial intelligence can also be applied to the green recycling of the supply chain (31), using artificial intelligence algorithms to optimize the layout of recycling points, the scheduling of recycling vehicles and the recycling path, so as to improve recycling efficiency and reduce recycling costs. At the same time, artificial intelligence technology is used to assess the damage degree and residual value of recycled products, so as to provide a decision-making basis for the reuse and disposal of recycled products. In short, as a technology with great potential, artificial intelligence provides a strong support for the development of green supply chain management practices. Through the application of green procurement, green production, green logistics, green sales and green recycling, artificial intelligence can improve the efficiency and effect of green supply chain management practices, and realize the optimal allocation of resources and environmental protection. Based on this, this paper puts forward the following hypothesis.

Hypothesis 2: AI positively affects green supply chain management practices.

### 2.3 Green supply chain management practice and sustainable development performance of public healthcare supply chain

The impact of green supply chain management practice on the sustainable development performance of public healthcare supply chain can be analyzed from the following three aspects. On the economic side, green supply chain management practices can reduce supply chain operating costs by optimizing production patterns and logistics distribution. As the society attaches importance to environmental protection, public medical supply chain that emphasizes green supply chain management practices is more likely to receive support and favor from the government and social capital, attract more high-quality suppliers to cooperate, enhance market competitiveness, and provide economic guarantee for sustainable development (32). At the social level, the practice of green supply chain management requires the public healthcare supply chain to strictly control the quality of medical products to ensure the safety and reliability of products entering the medical system, which helps to improve the safety and environmental protection of public medical services (33) and protect the health rights and interests of patients. Secondly, the public healthcare supply chain that actively practices green supply chain management can show a sense of responsibility to society and the environment, establish a good social image, enhance the public's trust and satisfaction with the public medical system, and promote the harmonious and stable development of society. At the environmental level, green supply chain management emphasizes the recycling of resources. In the public healthcare supply chain, strict recycling, cleaning, disinfection and reuse of reusable medical devices can reduce resource waste (34, 35), improve resource utilization efficiency and ensure the sustainability of medical services. At the same time, in the production of medical products, green supply chain management practices encourage enterprises to adopt green production technologies and processes, such as the use of environmentally friendly materials, sewage purification, etc., effectively reduce the discharge of pollutants such as wastewater, waste gas, waste residue, and reduce the negative impact on the environment. Based on this, this paper puts forward the following hypothesis.

Hypothesis 3: Green supply chain management practice positively affects the sustainable development performance of public healthcare supply chain.

### 2.4 The mediating role of green supply chain management practice

Green supply chain management practice means that enterprises integrate environmental issues into supply chain management practice (36) and implement green activities in all links of supply chain management. It covers the entire closing process of upstream green supplier procurement, enterprise internal environmental management and downstream green customer relationship (37), which is considered as an important business strategy for enterprises to reduce negative environmental impact and achieve profit and market share goals (38). Existing literature has studied the impact of green supply chain management practices on enterprise performance, and the research results show that green supply chain management practices are the reason for achieving sustainable competitive advantage of enterprises and the key to sustainable development of enterprises (39, 40). Faced with the shortage of resources and the aggravation of environmental pollution, green supply chain management activities are regarded as an important management strategy to improve sustainable development performance by the public healthcare supply chain. In the implementation of green supply chain management activities, the public healthcare supply chain actively incorporates artificial intelligence technology to optimize the production process and improve the waste discharge standards. Green supply chain management practice can not only reduce or prevent the production of pollutants, but also promote the public healthcare supply chain to achieve environmental protection standards, promote the integration and application of artificial intelligence in the public healthcare supply chain, and expand the application range of artificial intelligence technology in the public healthcare supply chain. In addition, AI has the advantage of optimizing supply chain operations, improving efficiency, and reducing waste (41). First, AI can be used in the analysis of big data to identify potential sustainability opportunities that exist in the public healthcare supply chain. Second, AI can also trace the quality of products throughout the supply chain to ensure compliance with environmental standards and regulations. Finally, when helping managers to make decisions, artificial intelligence is more inclined to recommend solutions that meet green environmental protection standards to decision-makers, which ensures the effective implementation of green supply chain management practices and is more conducive to the improvement of sustainable development performance of public healthcare supply chain. To sum up, the wider the coverage of artificial intelligence in the public healthcare supply chain, the greater the degree of implementation of green supply chain management practices, which plays an important role in reducing the waste of energy and resources in the production process and the negative impact on the environment, and then plays a positive role in improving the sustainable development performance of the supply chain. Based on this, hypothesis 4 is proposed.

Hypothesis 4: Green supply chain management practices play a mediating role between artificial intelligence and sustainable development performance of public healthcare supply chain.

### 2.5 The regulating effect of industry competition

The degree of industry competition covers two aspects of product homogeneity and price competition, which reflects the fierce degree of competition among enterprises in the whole industry. Competition in an industry is not only carried out among the original competitors, but there are five basic competitive forces: potential new entrants, competition of substitutes, bargaining power of buyers, bargaining power of suppliers, and competition among existing competitors (42). The status and comprehensive strength of these five basic competitive forces determine the intensity of competition in the industry, which determines the ultimate profit potential in the industry and the flow of capital to the industry, all of which ultimately determines the ability of enterprises to maintain high returns.

In the competition of product homogeneity, artificial intelligence can bring more heterogeneous resources to the supply chain, improve its competitive ability, and then obtain an advantageous competitive position. In addition, as a new production factor, artificial intelligence can optimize labor efficiency, thereby reducing supply chain production costs and improving operational efficiency (43), and further help the public healthcare supply chain achieve sustainable development. At the same time, existing research shows that when the industry competition becomes more intense, the digital capabilities of enterprises will become more powerful, which provides favorable conditions for the application of artificial intelligence. In addition, in order to alleviate the increasingly fierce industry competition and the uncertainty of the market environment, the public healthcare supply chain often tends to broaden the old business boundaries, and artificial intelligence can help enterprises identify market and economic data to help them expand more reasonably, thus improving the sustainable development performance of the supply chain.

To sum up, in a highly competitive industry, artificial intelligence technology has brought innovative opportunities and advantages to the development of enterprises, including improving operational efficiency, reducing production costs, increasing market share and sustainable development. Therefore, enterprises should flexibly use artificial intelligence technology to adapt to different competitive environments. Based on this, hypothesis 5 is proposed in this paper.

Hypothesis 5: Industry competition positively moderates the relationship between AI and sustainability performance in the public healthcare supply chain.

**Figure 1:**
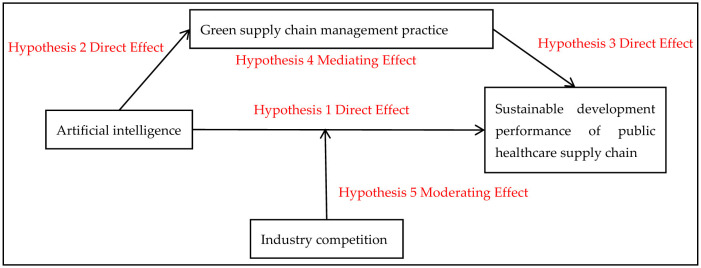
Theoretical model diagram.

In summary, the theoretical hypothesis model is established, as shown in Figure 1.

## 3 Results

### 3.1 Variable measurement

The variable measurement items used in this study are all from mature foreign research scales, and the Likert 5-level scale is used for measurement. On a scale of 1–5, respondents were asked to scale from “completely disagree” to “completely agree.” When completing the questionnaire, the interviewees are required to make corresponding choices based on their understanding of the company. In addition, according to the specific research situation, some variable measurement items were adjusted appropriately to ensure the accuracy of the meaning expression of the measurement items. The main concepts in this paper include artificial intelligence, green supply chain management practices, industry competition and supply chain sustainable development performance, and the corresponding measurement scales are shown in Table 1.

**Table 1:** Variable measurement scale.

**Variable**	**Measurement item**	**References**
Artificial intelligence (AI)	AI1: The extent to which your company uses AI technology	(3, 4, 18)
	AI2: Your company is able to use artificial intelligence technology to optimize supply chain operations	
	AI3: Your company is able to use AI technology for service/business innovation	
	AI4: Your company's artificial intelligent infrastructure network has a reasonable structure and high availability	
	AI5: Your company can acquire and master advanced artificial intelligence technology	
Green supply chain management practices(GSCM)	GSCM1: Your company has a clear environmental protection policy	(36–38)
	GSCM2: Your company adheres to green procurement and will give priority to green suppliers	
	GSCM3: Your company focuses on environmental management in the pharmaceutical manufacturing process	
	GSCM4: Your company has taken steps to reduce its carbon footprint during transportation	
	GSCM5: Your company has a systematic process to evaluate the environmental performance of your supply chain	
	GSCM6: Your company has implemented effective medical waste management	
	GSCM7: Your company regularly conducts staff training and publicity activities on environmental protection	
Industry competition(IC)	IC 1: In the past 3 years, your company has not experienced any failure of market competition law in protecting intellectual property rights	(42, 43)
	IC 2: Your company has a large market share	
	IC 3: Your company is in a market with a high growth rate	
	IC 4: Your company operates in a market with high barriers to entry	
	IC 5: Your products are highly differentiated	
	IC 6: Your company is actively developing new products or services	
Supply chain sustainability performance(SCSP)	SCSP1: Your company's energy consumption costs are reduced	(22–24)
	SCSP2: Your company's waste disposal costs are reduced	
	SCSP3: Reduce your company's use of toxic/hazardous materials	
	SCSP4: Your company's waste water, gas, solid waste discharge reduced	
	SCSP5: Your company has improved the working environment of its employees	
	SCSP6: Your company has improved employee safety	
	SCSP7: Your company improves the quality of life in the surrounding community	
	SCSP8: The frequency of environmental accidents in your company has decreased	

### 3.2 Sample selection and data collection

This study takes listed pharmaceutical enterprises in China as the research object. According to the research design of this paper and the review of existing literature, we adopt the form of questionnaire survey and invite middle and senior managers and backbone technical managers of pharmaceutical enterprises to answer. In order to improve the reliability of the sample data, a small scale pre-survey was conducted to verify the validity of the questionnaire, and then the questionnaire was modified accordingly to determine the final questionnaire. During the formal research stage from April 2024 to July 2024, a total of 320 questionnaires were collected, and 19 invalid questionnaires with repeated IP responses and inconsistencies were excluded. There were 301 valid questionnaires with an effective recovery rate of 94.06%, which met the requirements. The basic information of the samples is shown in Table 2. Among the sample enterprises, 31.6% of the respondents had college education or above, 30.9% had a bachelor's degree, and 37.5% had a master's degree or above. Among the respondents, middle and senior managers accounted for 46.1%, and technical managers accounted for 53.8%. The number of employees in the 100–500 accounted for 26.6%, 500–1,000 accounted for 24.9%, more than 1,000 accounted for 48.5%. Similarly, 14.0% of the enterprises have been operating for less than 10 years, and 86.0% of the enterprises have been operating for more than 10 years. These enterprises are in a relatively stable development cycle. Therefore, we believe that respondents can correctly understand the questions involved in the questionnaire and make objective choices, and the sample is representative to a certain extent.

**Table 2:** Describes the overall characteristics of the samples.

**Variable**	**Sample characteristics**	**Sample capacity**	**Percent**
Educational attainment	College and below	95	31.6%
	Bachelor degree	93	30.9%
	Master degree or above	113	37.5%
Enterprise scale	100–500 people	80	26.6%
	500–1,000 people	75	24.9%
	1,000–1,500 people	68	22.6%
	More than 1,500 people	78	25.9%
Position	Senior manager	29	9.6%
	Mid-level manager	110	36.5%
	Key technicians	162	53.8%
Years of business operation	Less than 10 years	42	14.0%
	10–20 years	32	10.6%
	20–30 years	115	38.2%
	More than 30 years	112	37.2%

### 3.3 Reliability and validity analysis

Firstly, the reliability analysis of the questionnaire was carried out. SPSS software was used to test the reliability of each observation index, and Cronbach's α values of 4 research variables were obtained, as shown in Table 3. Cronbach's α values of the four variables are all greater than 0.7, and CR values in the table are all greater than the acceptable threshold of 0.7, indicating that the sample has good reliability. Furthermore, SPSS software was used to conduct KMO and Bartlett sphericity test on the sample data, and the result was KMO = 0.942, p = 0.000 < 0.001 in the Bartlett sphericity test value, reaching a significant level. Generally speaking, when KMO > 0.8, sig. < 0.001 (some people also think sig. < 0.05), it indicates that there is a significant correlation between each item. The original data is suitable for factor analysis, so the sample data of this questionnaire is suitable for factor analysis. After factor analysis, the results show that the factor load of each item is greater than 0.7, indicating that the data has a certain representativeness and appropriateness, and the convergence validity meets the requirements. Thirdly, AMOS24.0 was used to conduct confirmatory factor (CFA) test. The research index standard was χ^2^/df was between 1 and 5, RMSEA was less than 0.05, CFI, NNFI and GFI were greater than 0.8 and greater than 0.9 was more standard. The fitting results of this model were as follows: χ^2^/df = 1.02, RMSEA = 0.008, CFI = 0.999, NNFI = 0.999, GFI = 0.942. Because each index met the requirements, the variable factor fit was high, indicating that the constructed model had good construction validity. In addition, the AVE values are all greater than the requirement of 0.5, and all are significant at the level. All the above fitting indicators meet the requirements, and the reliability and validity of this research model meet the requirements, with good internal quality.

**Table 3:** Factor loads and CR/AVE values of variables.

**Variable**	**Item**	**Factor loading**	**Cronbach's α coefficient**	**CR**	**AVE**
Artificial intelligence (AI)	AI1	0.783	0.887	0.887	0.612
	AI2	0.764			
	AI3	0.792			
	AI4	0.778			
	AI5	0.794			
Green supply chain management practices (GSCM)	GSCM1	0.772	0.918	0.918	0.615
	GSCM2	0.761			
	GSCM3	0.81			
	GSCM4	0.787			
	GSCM5	0.795			
	GSCM6	0.787			
	GSCM7	0.773			
Industry competition (IC)	IC1	0.766	0.908	0.908	0.623
	IC2	0.787			
	IC3	0.813			
	IC4	0.792			
	IC5	0.823			
	IC6	0.745			
Supply chain sustainability performance (SCSP)	SCSP1	0.8	0.929	0.929	0.62
	SCSP2	0.787			
	SCSP3	0.804			
	SCSP4	0.766			
	SCSP5	0.754			
	SCSP6	0.795			
	SCSP7	0.786			
	SCSP8	0.805			

All factor loading are significant.

### 3.4 Study 1 artificial intelligence positively affects the sustainable development performance of public healthcare supply chain

SPSS26.0 and Amos Graphics were used for reliability testing, confirmatory factor analysis and structural equation analysis to verify the proposed research hypothesis. The specific hypothesis contents and verification results are shown in Table 4. It is generally believed that the correlation is significant when p < 0.05. Therefore, the positive relationship between artificial intelligence and public healthcare supply chain is significant, and hypothesis 1 is supported.

**Table 4:** Hypothesis 1 test.

**Hypothesis**	**Path (structural model)**	**Path coefficient**	***P*-value**	**Conclusion**
Hypothesis 1	AI SCSP	0.405	0.000^***^	Hypothesis is true

*** Indicates a significant correlation at the 0.001 level (bilateral).

### 3.5 Study 2 AI positively affects green supply chain management practices

Structural equation model was used to test the relationship between artificial intelligence and green supply chain management practice. The specific hypotheses and verification results were shown in Table 5. It is generally believed that the correlation is significant when the path coefficient is greater than 0.4 and p < 0.05. Therefore, artificial intelligence has a positive impact on green supply chain management practices, and hypothesis 2 is assumed to be supported.

**Table 5:** Hypothesis 2 test.

**Hypothesis**	**Path (structural model)**	**Path coefficient**	***P*-value**	**Conclusion**
Hypothesis 2	AI GSCM	0.454	0.000^***^	Hypothesis is true

*** Indicates a significant correlation at the 0.001 level (bilateral).

### 3.6 Study 3 green supply chain management practice positively affects the sustainable development performance of public healthcare supply chain

Similarly, structural equation model is used to test the relationship between green supply chain management practice and sustainable development performance of public medical supply chain. The specific hypotheses and verification results are shown in Table 6. It can be seen from the data in the table that green supply chain management practices positively affect the sustainable development performance of public medical supply chain, assuming that hypothesis 3 is supported.

**Table 6:** Hypothesis 3 test.

**Hypothesis**	**Path (structural model)**	**Path coefficient**	***P*-value**	**Conclusion**
Hypothesis 3	GSCM SCSP	0.484	0.000^***^	Hypothesis is true

*** Indicates a significant correlation at the 0.001 level (bilateral).

### 3.7 Study 4 green supply chain management practices play a mediating role between artificial intelligence and sustainable development performance of public healthcare supply chain

As for the test of intermediary effect, existing studies usually refer to the causal stepwise regression method of Baron and Kenny (44). In recent years, many scholars have made improvements (45, 46). In this paper, the Bootstrap method in the Process program was used to sample 5,000 times to test the mediating effect of green supply chain management practice on the sustainable development performance of artificial intelligence and public medical supply chain. (1) To test whether the indirect effect interval contains 0: if it contains 0, the mediating effect is not significant and the test is stopped. If 0 is not included, the intermediary effect is significant, and the direct effect is tested. (2) If the mediating effect is significant, test whether the direct effect interval contains 0, if it contains 0, the direct effect is not significant and is completely mediating. Without 0, the direct effect is significant and partially mediated. The specific results are shown in Table 7.

**Table 7:** Conclusions of mediating effects.

**Path**	**Effect**	**Effect size**	**Boot standard error**	**95% confidence interval**	
				**BootCI lower bound**	**BootCI cap**
AI → GSCM → SCSP	Direct effect	0.214	0.054	0.108	0.321
	Indirect effect	0.147	0.029	0.091	0.203
	Total effect	0.359	0.053	0.255	0.463

The total effect of artificial intelligence on the sustainable development performance of public healthcare supply chain is 0.359, and 95% confidence interval is [0.255, 0.463], excluding 0, indicating that the total effect is significant. The direct effect of artificial intelligence on the sustainable development performance of public healthcare supply chain is 0.214, and the 95% confidence interval is [0.108, 0.321], excluding 0, indicating that the direct effect is significant. The indirect effect of artificial intelligence on the sustainable development performance of public medical supply chain through green supply chain management practice is 0.147, and the 95% confidence interval is [0.091, 0.203], excluding 0, indicating that the indirect effect is significant. In conclusion, the practice of green supply chain management plays a partial mediating role between artificial intelligence and sustainable development performance of public healthcare supply chain, and the hypothesis 4 is verified.

### 3.8 Study 5 industry competition positively moderates the relationship between AI and sustainability performance of public healthcare supply chain

The multi-level regression method in SPSS26.0 was used to test the moderating effect of industry competition between artificial intelligence and sustainable development performance of public healthcare supply chain. The results are shown in Table 8.

**Table 8:** Hierarchical regression analysis.

		**Sustainable development performance of public healthcare supply chain**			
		**M**_1_	**M**_2_	**M**_3_	**M**_4_
Control variable	Gender	−0.09	−0.08	−0.06	−0.06
	Age	0.03	0.05	0.05	0.05
	Educational attainment	0.08	0.04	0.04	0.05
	Enterprise scale	−0.11	−0.10	−0.09	−0.10
	Years of business operation	0.10	0.06	0.04	0.05
Independent variable	AI		0.36^***^	0.21^***^	0.19^***^
Regulating variable	Industry competition			0.37^***^	0.34^***^
Interaction term	AI × IC				0.19^***^
*R* ^2^		0.03	0.15	0.27	0.30
Adjusted *R*^2^		0.01	0.14	0.25	0.28
*F*		1.806	8.854	15.266	15.682

*** Indicates a significant correlation at the 0.001 level (bilateral).

As shown in Table 8, in order to test the regulatory effect of industry competition, the sustainable development performance of the public healthcare supply chain was taken as the dependent variable, artificial intelligence as the independent variable, and control variables were introduced to obtain the benchmark model M2 (in which M1 was taken as the benchmark model based on regression of control variables). On the basis of M2, the interaction terms of industry competition, artificial intelligence and industry competition are added, respectively, and M3 and M4 are obtained. The interaction term between AI and industry competition has a significant positive impact on the sustainability performance of public healthcare supply chain (M4, β = 0.19, *p* < 0.001). Hypothesis 5 has been verified that industry competition plays a positive moderating role between AI and sustainability performance of public healthcare supply chain.

**Figure 2:**
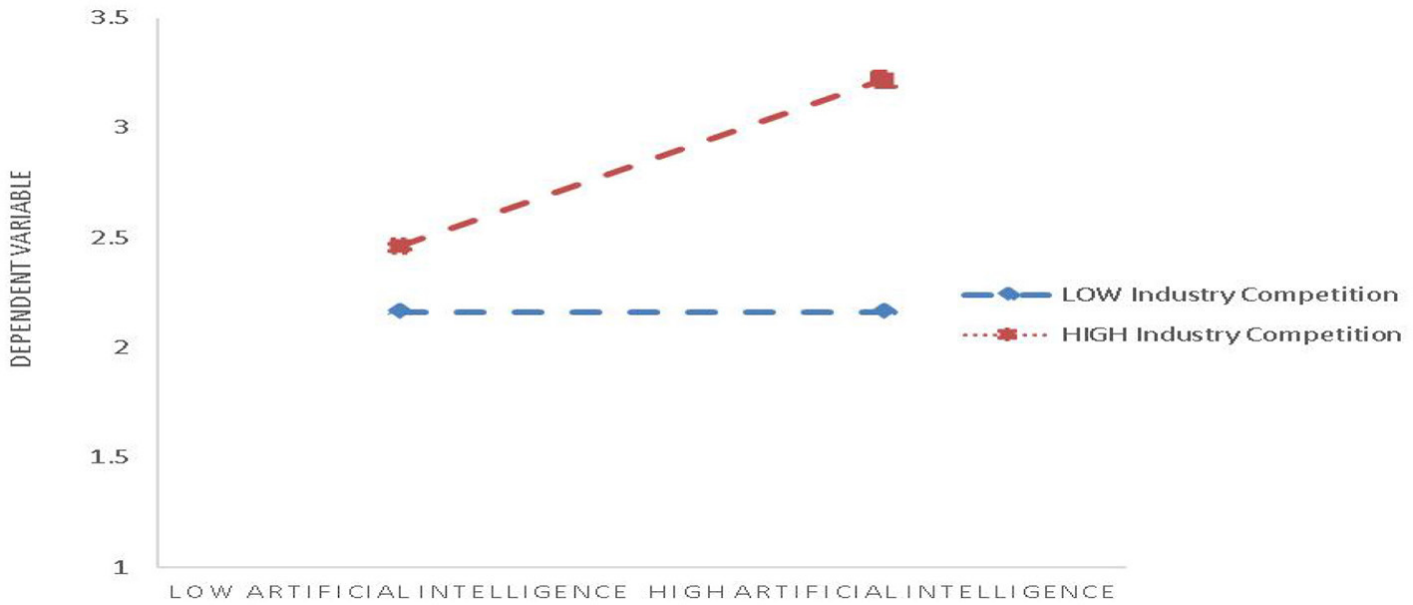
Moderating effect of industry competition on AI and sustainability performance of public healthcare supply chain.

In order to intuitively reflect the regulatory effect of industry competition, the regulatory effect chart is drawn at the baseline level 1 standard deviation above and 1 standard deviation below the mean of the regulatory variable. As shown in Figure 2. When industry competition changes from low value to high value, the slope of the effect of artificial intelligence on sustainable development performance of public healthcare supply chain becomes larger, indicating that the positive regulatory effect of industry competition is enhanced. It can be seen that in the supply chain with great influence on industry competition, artificial intelligence has a stronger positive impact on the sustainable development performance of the supply chain.

## 4 Discussion

Using SPSS26.0 and AMOS as tools, this paper discusses the relationship between artificial intelligence, green supply chain management practices, industry competition and sustainable development performance of public healthcare supply chain. Combined with the data collected from the questionnaire, this paper conducts an empirical study on the theoretical hypothesis model constructed, and draws the following conclusions: (1) Artificial intelligence has a significant positive impact on the sustainable development performance of the public medical supply chain, which indicates that medical enterprises can acquire digital capabilities through learning, realize the automation and intelligent management of operation processes, enhance their decision-making ability, and promote product innovation and service upgrading with the help of AI technology, so as to win market competitive advantages. Promote the sustainable development of enterprises. (2) Artificial intelligence positively affects the practice of green supply chain management, indicating the direction of sustainable development of the medical industry. Artificial intelligence helps the occurrence and implementation of green supply chain management practices, sets a benchmark for the industry, and promotes more enterprises to adopt relevant technologies to support the application of green supply chain management practices, which helps to unify the economic, social and environmental benefits of enterprises. (3) Green supply chain management practices positively affect the sustainable development performance of public medical supply chain, which clearly explains the role of green supply chain management practices in the sustainable development of enterprises. At the same time, it also reflects that the concept of environmental protection can be effectively combined with medical supply chain management. While pursuing the quality and efficiency of medical services, it may take into account the environmental benefits of enterprises, so as to realize the synergy of the two and help the sustainable development of the medical industry. (4) Green supply chain management practices play a partial mediating role in the sustainable development performance of artificial intelligence and public medical supply chain. Through the implementation of green supply chain management practices, artificial intelligence has changed the original single operation mode of pharmaceutical enterprises, and promoted the green transformation of the entire supply chain, which further clarified the social responsibility of enterprises. In addition, with the widespread application of artificial intelligence and green supply chain management practices in the medical supply chain, new policy guidance and technical standards may be generated, which will further regulate and guide the pharmaceutical industry to a smarter and more environmentally friendly direction. (5) Industry competition positively moderates the relationship between AI and sustainable development performance of public healthcare supply chain. In the highly competitive market environment, enterprises will seek and acquire new technologies more actively in order to maintain their market position, and the inclusion of artificial intelligence has become an important factor for enterprises to win in the competition. At the same time, with the increasing attention of consumers and regulators to environmental protection, enterprises may regard AI technology as one of the means of differentiation competition, in order to win the favor of consumers, so as to promote the sustainable development of enterprises. This study not only makes a theoretical contribution, but also has certain management significance. At the same time, we also point out the limitations of the study and the direction of future research.

## 5 Conclusions

This study has enriched the research on the relationship between artificial intelligence and sustainable development performance of public medical supply chain, and has strong theoretical significance, which is mainly reflected in the following three points: First of all, based on the influence mechanism of artificial intelligence, green supply chain management practice and industry competition on supply chain sustainability performance, this study deeply studies the relationship between these four factors, providing a new research idea for enterprises to build digital capabilities to improve sustainable development performance. Secondly, through a detailed discussion on the improvement process of sustainable development performance, this study has carried out a rich study on the path of competitive advantage improvement under the specific industry background. Previous studies have paid more attention to the direct relationship between digitalization and enterprise performance, and few have discussed its specific mechanism. Aiming at the gap of existing research, this study empirically studies the intermediary role of green supply chain management practice, and further expands the research ideas in this field. Finally, this study introduces industry competition as a moderating variable to explain that in supply chains with large industry competition influence, AI has a stronger positive impact on supply chain sustainability performance.

### 5.1 Implications

The conclusion of this study has important reference significance for pharmaceutical enterprises to improve sustainable development performance. First, attention should be paid to the positive impact of artificial intelligence on the improvement of supply chain sustainability performance. On the one hand, it is necessary to enrich digital consciousness, information is not equal to digitization, and it is necessary to invest in ideas, methods, technologies and means that are different from traditional element resources to mine data value. In terms of artificial intelligence software, all links of the supply chain can be optimized by digital technology. In terms of artificial intelligence hardware, cutting-edge technologies such as unmanned driving, AI algorithms, intelligent Internet of Things technology, etc. can be implemented into actual operation scenarios to help improve the operational efficiency of the supply chain. On the other hand, choose the path suitable for its own development strategy, and use the power of artificial intelligence to improve the enterprise's data collection, data analysis and application capabilities, so that the supply chain business can be digitized, visualized and standardized. Second, give full play to the advantages of green supply chain management practices to lay the foundation for sustainable development. Taking green supply chain management practices as a bridge, on the one hand, enhancing the effect of the application of artificial intelligence in supply chain management, on the other hand, through green supply chain management practices, enterprises can reduce waste emissions and reduce the damage to the environment in the operation process, so as to achieve a higher level of sustainable development goals. Third, industry competition is one of the important boundary conditions for enterprises to improve sustainable development performance through AI. On the one hand, pharmaceutical companies can cultivate their own unique market competitiveness, and use artificial intelligence technology faster, better and more creatively in the future to adapt to the trend of industrial change; On the other hand, a special market analysis group can be set up to adjust the use of artificial intelligence technology within the enterprise, constantly search for the best resources and formulate development plans suitable for the enterprise itself, so as to pave the way for innovative development.

### 5.2 Limitations and future research

First of all, the data in this paper are obtained from A questionnaire survey, which cannot fully reflect the extent of the use of artificial intelligence by enterprises, and the measurement criteria have limitations. Therefore, in the subsequent research, a more scientific way can be adopted to evaluate indicators, such as the verification and analysis of the methods of A and B papers. Secondly, in addition to industry competition, there are other factors worthy of further discussion and research in the selection of model adjustment variables. At the same time, whether the industry competition has intermediated regulation is also worth further research.

In conclusion, this paper deepens the study of artificial intelligence on the sustainable development performance of public medical supply chain, solves the technical bottleneck faced by existing research, compensates the content defects of existing research, optimizes the operation process of public medical supply chain, and verifies the mediating role of green supply chain management practice and the regulating role of industry competition. It provides novel research ideas for the long-term development of public health care and innovative theoretical and methodological support for the sustainable development of supply chain.

## Data Availability

The raw data supporting the conclusions of this article will be made available by the authors, without undue reservation.
